# Validation and Clinical Utility of a Prediction Model for the Risk of Upstaging to Invasive Breast Cancer After a Biopsy Diagnosis Ductal Carcinoma In Situ

**DOI:** 10.1245/s10434-023-13929-y

**Published:** 2023-08-04

**Authors:** Claudia J. C. Meurs, Sara van Bekkum, Joost van Rosmalen, Marian B. E. Menke-Pluijmers, Sabine Siesling, Pieter J. Westenend

**Affiliations:** 1 Department of HTSR, University of Twente, Enschede, The Netherlands; 2 CMAnalyzing, Zevenaar, The Netherlands; 3 Department of Surgery, Albert Schweitzer Hospital, Dordrecht, The Netherlands; 4 Department of Biostatistics, Erasmus Medical Centre, Rotterdam, The Netherlands; 5 Department of Epidemiology, Erasmus Medical Centre, Rotterdam, The Netherlands; 6 Department of Research, Netherlands Comprehensive Cancer Organisation, Utrecht, The Netherlands; 7 Laboratory of Pathology Dordrecht, Dordrecht, The Netherlands

## Abstract

**Background:**

This study aimed to validate the DCIS-upstage model, a previously developed model to predict the risk of upstaging to invasive breast cancer in patients with biopsy-proven ductal carcinoma in situ (DCIS) in a more recent cohort and to assess the model’s clinical utility.

**Methods:**

The model was validated in a registry cohort (*n* = 2269) and in an institution cohort (*n* = 302). A calibration plot was made, followed by a decision curve analysis (DCA). The model’s area under the curve (AUC) was compared with the AUC of another published model and with the AUCs of new models using the risk factors of the DCIS-upstage model and additional risk factors.

**Results:**

The DCIS-upstage model had an AUC of 0.67 at development; in the validation, the AUC was 0.65 in the registry cohort and 0.73 in the institution cohort. The DCA showed that the model has clinical utility. The other published model had an AUC of 0.66 in the institution cohort. Adding risk factors to the DCIS-upstage model slightly increased the AUC.

**Conclusions:**

The DCIS-upstage prediction model is valid in other cohorts. The model has clinical utility and may be used to select patients with biopsy-proven DCIS for sentinel lymph node biopsy.

In the Netherlands, each year approximately 2300 patients are diagnosed with ductal carcinoma in situ (DCIS) of the breast. Although the initial diagnosis is usually based on biopsy, the diagnosis may be upstaged to invasive breast cancer following pathologic examination of the surgical specimen.^1^ In a previous study, we showed an upstaging rate of 21% for patients diagnosed in 2011 and 2012 in the Netherlands.^2^ While axillary staging is indicated in patients with invasive breast cancer, the role of axillary staging in patients with biopsy-proven DCIS is debated. In case of upstaging to invasive breast cancer, axillary staging may alter treatment.^2^ In several countries, such as the United Kingdom and the Netherlands, the decision to perform a sentinel lymph node biopsy (SLNB) in patients with biopsy-proven DCIS is partly based on the presence of risk factors such as the histological grade of the biopsy-proven DCIS.^3,4^

At the individual patient level, the risk of upstaging to invasive breast cancer after biopsy-proven DCIS can be estimated with the DCIS-upstage model (https:/Evidencio.com/models/show/1074), a population-based prediction model developed by our research group.^2^ This model includes information on the detection mode, the palpability of the lesion, the histologic grade of the DCIS at biopsy, histologic suspicion on the presence of an invasive component at biopsy, and the BI-RADS score on mammography.^2^ In the Netherlands, all this information is routinely collected during diagnostic workup. Other studies have also developed prediction models for upstaging after biopsy-proven DCIS.^5–13^ For instance, Jakub et al. developed a prediction model on the basis of the DCIS grade on core needle biopsy, the presence of a mass on imaging, multicentricity of the lesion, and the largest linear dimension on mammography.^7^

A few validation studies of models were published, which showed that the prediction models performed statistically slightly worse in the validation cohorts than in the patient population in which the model was developed.^7,8^ Comparison of the available models is complicated by the large variety of risk factors that are used in prediction models. Furthermore, comparison of the models is not always fully possible because some studies did not report the intercept of their model.^5,6,12,13^ Furthermore, the comparison of the different models is complicated by the differences in the average upstaging rates. Within the populations on which the models were developed, the rates varied from 14% to 37%.^7,8^ This variation might not only be due to differences in the selection of patients but also to differences in diagnostic workup between hospitals or countries or over time. When using a prediction model, it therefore is important that the cohort has some similarity with the model development cohort.

The aim of our study was to validate our DCIS-upstage model in a more recent patient cohort, to assess the model’s clinical utility, compare the model’s performance with the performance of another published prediction model developed by Jakub, and to determine the effect of adding other risk factors to the prediction model.

## Patients and Methods

### Study Design and Population

This study used two cohorts, a registry cohort (*n* = 2269) and an institution cohort (*n* = 302). The registry cohort is representative of the entire population. Due to the large sample size of the registry cohort, this cohort was best suited for validating and assessing the clinical utility of our previously developed prediction model for the risk of upstaging, which in this study is referred to as the DCIS-upstage model.^2^ The institution cohort was used to compare the model with the model published by Jakub et al.^7^ because that cohort contains data that are not available in the registry cohort. The Jakub et al. model uses tumor data that are normally available in hospitals in the Netherlands.^7^ Both models are available online; see https://www.evidencio.com/models/show/1074 and https://www.evidencio.com/models/show/950. The institution cohort was also best suited for analyzing the effects of extending the DCIS-upstage model with additional risk factors.

Data of the registry cohort were collected from the Dutch Pathology Registry, which is managed by PALGA, the Dutch Pathology Databank of the Netherlands. The registry contains all the reports of material examined by pathologists in Dutch Pathology Laboratories.^14^ For all patients, reports were available on cytology, biopsies, lymph node biopsies, and excisions. Patients registered in the pathology registry were matched to patients in the Netherlands Cancer Registry (NCR), which is hosted by the Netherlands Comprehensive Cancer Organization (IKNL). This step resulted in a combined dataset with the following data from PALGA: age at the time of biopsy, presence of contralateral DCIS or invasive breast cancer, DCIS grade at biopsy, suspicion on presence of an invasive component at biopsy, histopathologic microcalcification, and comedonecrosis. The NCR data included mode of detection, palpability, BI-RADS score, a history of malignancy, contralateral tumors, clinical and pathological *T* and *N* status, surgical treatment information, and final diagnosis.

Needle biopsies with a diagnosis of DCIS were selected if they were reported in a standardized structured pathology protocol between July 2016 and March 2019. In case of more than one biopsy with the diagnosis DCIS, the biopsy with the most tumor information was selected. In case the report stated any doubt about the diagnosis DCIS, the patient was not included. Patients were excluded if they had an ipsilateral history of DCIS or invasive breast cancer, since the current diagnosis could be either a recurrence or a second tumor. Patients were also excluded if they had proven positive lymph nodes or microinvasion at the time of biopsy, since these patients were considered to have invasive breast cancer. Moreover, patients were excluded if the excision was performed more than 3 months after the biopsy that proved DCIS. Finally, patients were excluded for whom not all the information that was used in the DCIS-upstage model was available.

The institution cohort comprised all consecutive patients diagnosed with DCIS from July 2012 to December 2018 at the Albert Schweitzer hospital in the Netherlands. Data were retrieved from the electronic medical records of the institution and from the pathology records of the Laboratory of Pathology Dordrecht. The available data included age, detection mode, referral reason, family history of malignant disease, a history of a malignancy, contralateral tumors, palpability, BI-RADS score, mass on mammography, size on mammography, multicentricity across quadrants on mammography, multifocality within quadrants on mammography, calcification on mammography, density, estrogen receptor (ER) status, HER2 neu receptor status, DCIS grade at biopsy, type of first resection, and final diagnosis. Patients were excluded from the institution cohort if not all the information for them that was used in the DCIS-upstage model and the Jakub prediction model was available.

Upstaging was defined as a diagnosis of microinvasive or invasive breast cancer found in the surgical specimen while the diagnosis at biopsy had been DCIS. If lymph nodes were positive but the excision diagnosis remained DCIS, the patient was not considered being upstaged. Contralateral breast cancer was defined as DCIS or invasive breast cancer in the other breast, previously or in the same period as the biopsy-proven DCIS. In case of the presence of multiple DCIS grades or any doubt about the histologic grade, the highest grade that was mentioned was used. Suspected invasive component was coded as “yes” if the pathologist specifically mentioned that there was a suspicion of invasion or if invasion could not be ruled out by additional immunohistochemistry. In both cohorts no data on race or ethnicity were collected. In the Netherlands, 74% of residents and both parents of resident are born in the Netherlands.^15^

### Statistical Analyses

For both cohorts, patient and tumor characteristics were compared between patients with and without upstaging to invasive breast cancer using the Mann–Whitney U test for continuous data and the chi-squared test or the Fisher’s exact test for categorical data. For the institution cohort, associations between risk factors were analyzed with the chi-squared test or Fisher’s exact test.

The DCIS-upstage model was validated in both cohorts and the Jakub model was validated in the institution cohort using the logistic regression model. A calibration analysis was performed. Results were evaluated with the area under the curve (AUC) of the receiver operating characteristic (ROC) curve and with the slope and intercept of the calibration plot, as well as by assessing the calibration for risk categories. For the ROC curve, the maximum Youden’s index was calculated, and for the corresponding probability, cut off the sensitivity, specificity, positive predictive value, and negative predicted value. It was decided whether re-estimation of the risk factors was needed to accommodate for differences over time by comparing the intercept of the calibration plot with the intercept when both the intercept and the slope were re-estimated. In the institution cohort, patients were grouped on the basis of predicted risk, and the observed upstage rate was assessed in each group.

The ratio of true positive to false positive predictions was calculated in the decision curve analysis (DCA) and is reflected in a net benefit. At a range of cut-off points, the net benefit of the model was calculated, as was the net benefit when assuming that all patients had invasive breast cancer and the net benefit when assuming that none of the patients had invasive breast cancer. At cut-off points at which the net benefit of the model was higher than the benefit calculated if all or no patients had invasive breast cancer, the model was considered to be clinically useful.

New models were made using the variables of the DCIS-upstage model and extending the model with additional risk factors. The additional variables were the ER/HER2 category or mass and size on mammography. For the analyses, all beta coefficients of the risk factors of the DCIS-upstage model were automatically re-estimated. The AUC of the new models was compared with the AUC when using only the risk factors of the DCIS-upstage model.

Pearson correlations of the predicted risk per patient were analyzed between the DCIS-upstage model and the model of Jakub. The model of Jakub was also re-estimated, and the correlation between the re-estimated model of Jakub and the re-estimated DCIS-upstage model was analyzed, as was the correlation between the re-estimated model of Jakub and the original model of Jakub and between the re-estimated DCIS-upstage model and the original DCIS-upstage model.

## Results

### Registry Cohort

In total, 127 (5.3%) patients from the registry cohort were excluded that had missing data for one or more of the risk factors that were used in our prediction model; 21 missing values for detection mode, 60 for palpable, 14 for DCIS grade, and 54 for BI-RADS score.

Thereafter, the registry cohort was made up of 2269 patients with a median age of 59 years, and 34% were younger than 55 years. The detection mode was screening in 67% of the patients, and the tumor was palpable in 17%. The DCIS grade was low in 12%, intermediate in 42% and high in 46% of the patients. The BI-RADS score was 1–3 in 5%, 4 in 86%, and 5 in 9%, and 2% were suspected to have invasive breast cancer.

The upstaging rate was 17.4% (*n* = 395). The upstaging rates for the risk factors that are included in the DCIS-upstage model are presented in Table 1. Associations between other patient and tumor characteristics are shown in Appendix A.

**Table 1:** Patient and tumor characteristics: comparison of upstaged patients (*n* = 395) and non-upstaged patients (*n* = 1874) of the registry cohort

	All patients	Upstaging to invasive breast cancer				
		No		Yes		*p*-value*
	*N*	*N*	%	*N*	%	
Number (%)	2269	1874	83%	395	17%	
Detection mode						< 0.001
Screening	1530	1313	86%	217	14%	
Otherwise	739	561	76%	178	24%	
Palpable						< 0.001
No	1894	1618	85%	276	15%	
Yes	375	256	68%	119	32%	
DCIS grade						< 0.001
Low	263	238	90%	25	10%	
Intermediate	954	794	83%	160	17%	
High	1052	842	80%	210	20%	
BI-RADS score						< 0.001
3	117	99	85%	18	15%	
4	1939	1641	85%	298	15%	
5	213	134	63%	79	37%	
Suspected invasive component at biopsy						0.001
No	2221	1843	83%	378	17%	
Yes	48	31	65%	17	35%	

*Comparison between upstaged and non-upstaged patients using the chi-squared test

**Fig. 1:**
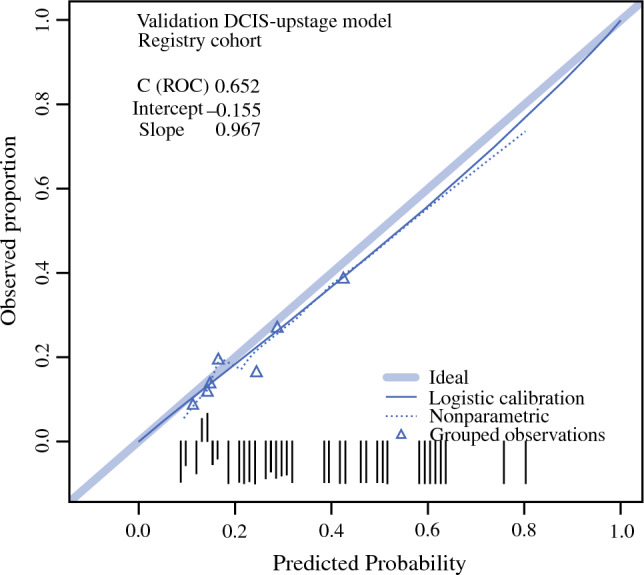
Calibration plot of the DCIS-upstage model on the registry cohort (*n* = 2269): the grouped observation represents groups of 10% of the patients; a logistic and a nonparametric line is drawn through the grouped observation; the groups will be plotted on the ideal line if the predicted probability is equal to the observed proportion

The predicted probability of upstaging was plotted against the observed proportion of upstaging in the calibration plot (Fig. 1). The intercept was −0.155, the slope 0.967, and the *C*-statistic (AUC) of the ROC curve was 0.65. The overestimation was 15.5%, which was not significant (*p* = 0.267). The ROC curve is shown in Appendix B.

**Fig. 2:**
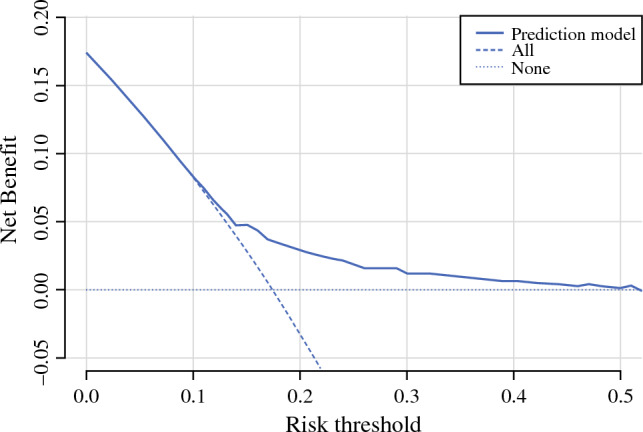
DCA of the DCIS-upstage model on the registry cohort (*n* = 2269)

The DCA of the model showed that the model provides a net benefit (compared with a “treat all” strategy) if the risk threshold is higher than 10%, and that the net benefit becomes greater if the risk thresholds are higher than 14%. The net benefit again becomes close to 0 at risk thresholds of 40% or higher, due to a very limited benefit compared with a “treat none” strategy (Fig. 2). In this validation cohort, the mean predicted risk was 18.9%, ranging from 9.5% to 80.2% with a median of 14.7%. In the cohort, 249 patients (11%) had a risk below 14%, 1907 (84%) patients had a risk between 14 and 40%, and 113 (5%) had a risk of more than 40%.

### Institution Cohort

In total, 8 (2.6%) patients of the institution cohort were excluded that had missing data for one or more of the risk factors that were used in our prediction model; 6 missing values for palpable, 2 for DCIS grade, and 2 for multicentric.

Thereafter, the institution cohort consisted of 302 patients whose upstaging rate was 20.2%. Table 2 presents the associations between the upstaging rate and the variables that were used in the models. The associations of upstaging with other variables that were explored are shown in Appendix C. Upstaging was significantly associated with reason of referral, palpability, presence of mass on mammogram, breast density, lesion size, BI-RADS score, presence of calcifications, multifocality, DCIS grade, and a suspected invasive component at biopsy.

**Table 2:** Patient and tumor characteristics: comparison of upstaged patients (*n* = 61) and non-upstaged patients (*n* = 241) of the institution cohort

	All patients	Upstaging to invasive breast cancer				
		No		Yes		*p*-value^*^
	*N*	*N*	%	*N*	%	
Number (%)	302	241	80%	61	20%	
Age in years Mean, median (range)	58.8, 58 (28–88)	58.8, 58 (33–82)		58.9, 59 (28–88)		0.912^**^
Detection mode						0.010^#^
Screening	187	158	84%	29	16%	
Otherwise	115	83	72%	32	28%	
Palpable						< 0.001^$^
No	250	214	86%	36	14%	
Yes	52	27	52%	25	48%	
Mass on mammography						< 0.001^#^
No	235	204	87%	31	13%	
Yes	67	37	55%	30	45%	
BI-RADS score						< 0.001^$^
3	4	4	100%	0	0%	
4	271	225	83%	46	17%	
5	27	12	44%	15	56%	
Size (mm) Mean, median (range)	22, 15 (2–100)	19, 13 (2–100)		33, 26 (4–100)		< 0.001^**^
Multicentric across quadrants						0.744^$^
No	287	228	79%	59	21%	
Yes	15	13	87%	2	13%	
DCIS histological grade at biopsy						0.038^$^
Low	53	47	89%	6	11%	
Intermediate	70	60	86%	10	14%	
High	179	134	75%	45	25%	
Suspected invasive component at biopsy						< 0.001^$^
No	286	235	82%	51	18%	
Yes	16	6	38%	10	63%	
ER/HER2 category						0.054^$^
ER positive and HER2 negative	162	135	83%	27	17%	
Other	132	98	74%	34	26%	
Missing	8	8	100%	0	0%	

*p-Value of comparison between upstaged and non-upstaged patients determined with the Mann–Whitney U test**, the chi-squared test^#^, or Fisher’s exact test^$^

Calibration of the Jakub model showed that the intercept was −0.166, with a slope of 0.635, and the AUC of the ROC was 0.66. Appendix D shows the calibration plot and ROC curve of the model by Jakub and the calibration plot and ROC curve of the DCIS-upstage model on the institution cohort. The correlation between the predicted risks of the DCIS-upstage model and of the Jakub model was 0.23. The correlation was again analyzed after re-estimating the beta coefficients of each risk factor, resulting in a correlation of 0.67. The correlation between these re-estimated models and the original models was 0.50 for the Jakub model and 0.96 for the DCIS-upstage model.

**Fig. 3:**
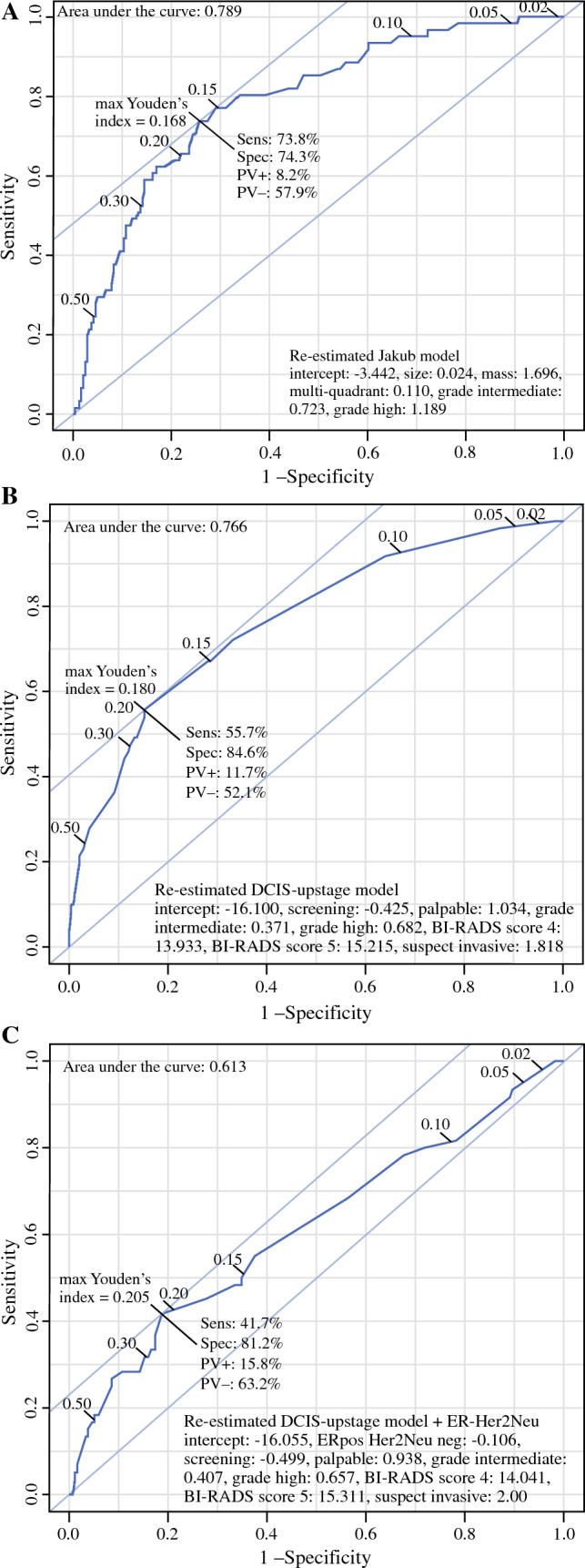

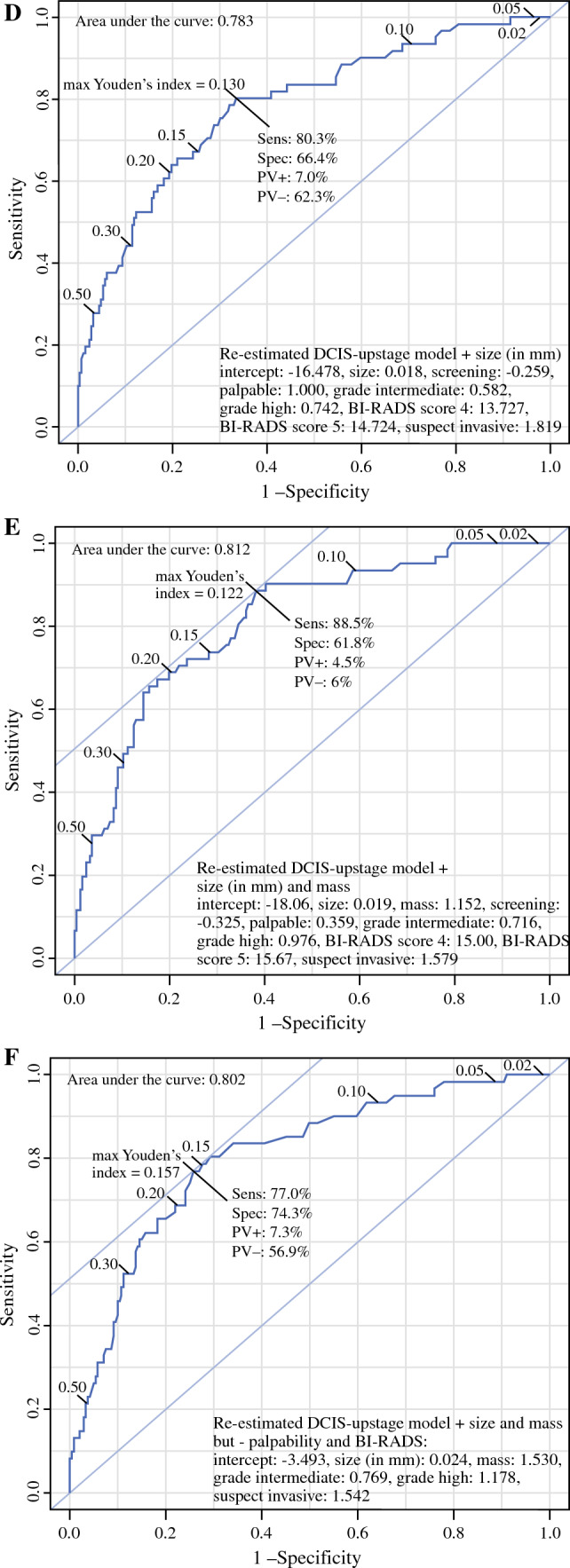
ROC curves of the DCIS-upstage model, the Jakub model, and the re-estimated and extended models, calculated on the institution cohort, *n* = 61 upstaged patients; ROCs of the re-estimated models on the institution dataset: **a** Jakub model, **b** DCIS-upstage model, **c** DCIS-upstage plus ERHer2neu, **d** DCIS-upstage plus size, **e** DCIS-upstage plus size and mass, and **f** DCIS-upstage plus size and mass minus palpable and detection mode; the figures indicate the intercept, the beta-coefficients, and the AUCs of the models

Figure 3 shows the ROC curves of the re-estimated model of Jakub and the re-estimated DCIS-upstage model and the ROC curves of the extended DCIS-upstage models. After re-estimation, the model of Jakub had an AUC of 0.79 and the DCIS-upstage model had an AUC of 0.77. Several models were made by adding risk factors to the DCIS-upstage model (Fig. 3c–f). The model with the highest AUC (0.81) comprised the variables detection mode, palpability, DCIS grade at biopsy, a suspected invasive component at biopsy, size of the lesion at mammography, and presence of mass on mammography (see Fig. 3e). Addition of the ER/HER2neu variable did not improve the model. Analysis of the associations showed that ER/HEr2neu was strongly associated with the DCIS grade (*p* < 0.001); in ER positive/HER2 neu negative patients the DCIS grade was 30% low, 30% intermediate, and 40% high grade. In patients with other receptor combinations the DCIS grade was 3% low, 13% intermediate, and 84% high grade. Patients with ER positive and HER2neu negative DCIS also had a lower palpability rate, 13% versus 24% (*p* = 0.019), and a lower rate of mass on mammography, 17% versus 30% (*p* = 0.013), compared with other receptor combinations.

The predicted risk for upstaging using the DCIS-upstage model ranged from 10.8% to 80.2%, with a median of 16.8% and mean of 20.4%. Dividing the patients into three groups on the basis of predicted risk (group 1 < 15%, group 2 ≥ 15–< 25%, and group 3 ≥ 25%) resulted in group 1 with 99 patients (33%), of whom 12 were upstaged (12.1%); group 2 with 139 patients (46%), of whom 18 were upstaged (13.0%); and group 3 with 64 patients (21%), of whom 32 were upstaged (48.4%). In group 1, SLNB was performed in 109 patients (62%) and two macrometastases were found, in group 2 SLNB was performed in 39 patients (67%) and two micrometastases were found, and in group 3 SLNB was performed in 61 patients (91%) and four micrometastases and three macrometastases were found.

## Discussion

In this study we validated the DCIS-upstage model on a new cohort and assessed the clinical utility of the model. We also compared the model with another published prediction model and evaluated the impact of extension of our prediction model with additional risk factors. Evaluation of the fit of the model showed a relative overestimation by 16% on the predicted risk. The AUC was almost identical to the AUC of the original model. The model had clinical utility when using cut-off points of the predicted risk of 14% or higher. The model performed comparable to the Jakub model. Adding other risk factors to the model increased the AUC with 0.04.

Although the average upstaging risk has decreased over time, the model was valid in a new cohort. The average upstaging risk was 17.4% in the registry cohort compared with 20.6% in the cohort in which we developed the DCIS-upstage prediction model.^2^ The model calibration plot showed that the observed proportion of upstaging was lower than the predicted proportion in the patient groups with a high predicted risk; this was somewhat more than in the patient group with a low predicted risk. However, the overestimation was not statistically significant, and therefore re-estimation of the model was not required.

The model can be used to select patients for a SLNB on the basis of their individual risk of upstaging. However, it is important to bear in mind that each prediction model will result in true positive and false positive predictions. The ratio between them differs for each cut-off point that is chosen. When setting the cut-off point of the model very low, many patients will be selected for SLNB and this will result in many false positive predictions, and setting the cut-off very high will result in many false negative predictions. In a clinical decision analysis, we demonstrated that a cut-off point between 14% and 40% should be selected. For instance, in practice in our institution, SLNB was performed in 69% of patients (209 patients). In the case that patients would have been selected with the DCIS-upstage model with a threshold of 25%, only 22% of the patients (67 patients) would have been selected for SLNB and the upstage rate would have been 48% (32 patients). For patients that would not have been selected for SLNB, the upstage rate would have been 12% (29 out of 235 patients). The changes in the percentage of patients that are selected for SLNB do not solely depend on the threshold chosen. In the Netherlands SLNB is considered for all patients. In some countries SLNB is only considered in patients undergoing mastectomy and is omitted in the case of breast-conserving surgery. It should be kept in mind that most often in the case of breast conserving surgery, a secondary SLNB can be done, and in the case of mastectomy, secondary SLNB is practically impossible. The disadvantage of having a second operation should be weighed against selecting patients for SLNB on the basis of our model.

Prediction models can be built with a wide range of risk factors, all resulting in comparable discriminative strength. The model of Jakub et al. uses DCIS grade, mass, multicentricity, and size, whereas we use DCIS grade, detection mode, palpability, BI-RADS score, and a suspicion of invasiveness in biopsy specimens. The correlation between both models was low, resulting in discordant predictions. The correlation could be improved by re-estimating the models to the institution cohort. Re-estimation of the Jakub model changed the risk of size most, and size was a more predictive risk factor in the institution cohort than in the Jakub cohort.

In the current validation study, the AUC was 0.65 in the registry cohort and 0.73 in the institution cohort. This was comparable with the AUC of 0.67 in the study in which we developed our prediction model. Multiple prediction models for upstaging have been developed. Validation studies of these models compared the AUC of the development cohort with the validation cohort. In the study of Lee et al. the AUC was 0.82 and 0.70,^9^ respectively, in the study of Park et al. it was 0.76 and 0.72,^11^ respectively, in the study of Coufal et al. it was 0.76 and 0.85,^10^ respectively, and in the study of Jakub et al. it was 0.73 and 0.71,^7^ respectively. Jakub et al. validated the other models in their study cohort and found AUCs of 0.59 for the study of Lee, 0.63 for the study of Park, and 0.66 for the study of Coufal.^7^ In our cohort, the Jakub model had an AUC of 0.66. Overall, it seems that the AUCs of different prediction models are of the same order size and become somewhat lower in other cohorts as compared with the development cohort. This might be due to patient selection in the model development process. In addition, differences between countries in the diagnostic process and techniques of biopsy and imaging might influence the discriminative ability of the model. The increase in the AUC of the Jakub model from 0.66 to 0.79 by adapting the coefficients of the model to our institution cohort might indicate the effect of those differences. This increase could be partly due to overfitting of the model. Adapting the coefficients for our own model only resulted in a change in AUC from 0.73 to 0.77, indicating that the impact of an overfit of the model is limited.

Adding variables to the DCIS-upstage model improved the AUC of the model slightly. The combination of ER receptor status and HER2neu status is considered an important factor for the risk of invasive breast cancer.^9,16^ However, adding the combined ER/HER2neu status to our model did not increase the discriminative ability of the model. In this study the ER/HER2 neu status was associated with DCIS grade, palpability, and mass. However, in univariable analysis, ER/HER2 neu was also not significantly associated with upstaging (*p* = 0.054), thus it was expected that in multivariable analyses the receptor status would not improve the model. Mass and size are risk factors that are used in several other models.^6,7,9–11^ Adding mass and size to our model resulted in an increase of 0.04 of the AUC, from 0.77 to 0.81, in the institution cohort. Compared with our developed model, the sensitivity of that model is increased, however, the specificity is decreased. Before using this model, validation is needed first.

Applying the DCIS-upstage model must be carried out with caution. In our study, we did not select patients with microinvasive cancer at biopsy, patients who underwent excisional biopsy, and patients with a history of ipsilateral DCIS or invasive breast cancer. For these patients, the prediction model for upstaging is not applicable.

A limitation of this retrospective study was that information on the diagnostic workup in the registry cohort was sparse and therefore not all potentially useful risk factors were recorded in the registry cohort: for example, information on mammography and on the size of biopsy needle is lacking, although it is reasonable to assume that the vast majority of the biopsies were vacuum assisted. A strength of this study is that we had a second cohort: an institutional cohort with many additional clinical variables such as information on imaging. Another strength of this study was that the validation was done on the registry-based cohort, which was a large cohort and representative of daily practice as well.

The next research step would be to assess the effect of implementation of this model on the accuracy of the use of the SLN procedure in patients with biopsy-proven DCIS.

## Conclusions

This study confirmed the validity of our previously developed model for upstaging. This prediction model for upstaging has clinical utility and may be used in the selection for SLNB in patients with biopsy-proven DCIS.

## Appendix A

Upstaging rate in the registry cohort: associations in the registry cohort of patient and tumor characteristics with upstaging to invasive breast cancer

**Table:**

	All patients	Upstaging to invasive breast cancer				
		No		Yes		*p*-Value*
	*N*	*N*	%	*N*	%	
Number (%)	2269	1874	83%	395	17%	
Year of incidence						0.541^#^
2016	261	220	84%	41	16%	
2017	820	685	84%	135	17%	
2018	957	778	81%	179	19%	
2019	231	191	83%	40	17%	
Age, mean, median (range)	59.4, 59 (23–90)	59.7, 60 (23–90)		58.3, 58 (26–88)		0.024^**^
Age categories						0.030^#^
< 55	778	624	80%	154	20%	
≥ 55	1491	1250	84%	241	16%	
Microcalcification						< 0.001^$^
Not present	350	263	75%	87	25%	
Present	1909	1602	84%	307	16%	
Missing	10	9	90%	1	10%	
Comedonecrosis						0.007^#^
Not present	492	406	83%	86	17%	
Present	1368	1109	81%	259	19%	
Missing	409	359	88%	50	12%	
cT						< 0.001^$^
In situ	2201	1868	85%	333	15%	
1	35	0	0%	35	100%	
2–4	18	0	0%	18	100%	
Missing	15	6	40%	9		60%
cN						0.004^$^
0	2251	1863	83%	388	17%	
1	3	0	0%	3	100%	
Missing	15	11	73%	4	27%	
Contralateral						0.063^#^
No	2235	1850	83%	385	17%	
Yes	34	24	71%	10	29%	

**p*-Value of comparison between upstaged and non-upstaged patients determined with the Mann–Whitney U test^**^, the chi-squared test^#^, or the Fisher’s exact test^$^

## Appendix B

ROC curve of the DCIS-upstage model: ROC curve of the model for upstaging to invasive breast cancer, on the registry cohort; the maximum Youden’s index was 0.1479

## Appendix C

Upstaging rate in the institution cohort: associations in the institution cohort of patients and tumor characteristics with upstaging to invasive breast cancer

**Table:**

	All patients	Upstaging to invasive breast cancer				
		No		Yes		*p*-Value*
	*N*	*N*	%	*N*	%	
Number (%)	302	241	80%	61	20%	
Age categories						0.948^$^
< 55 years	110	88	80%	22	20%	
≥ 55 years	192	153	80%	39	20%	
Family history of mamma disease						0.822^$^
No	179	145	81%	34	19%	
Yes	95	74	78%	21	22%	
Missing	28	22	79%	6	21%	
Referral reason						< 0.001^$^
Screen-detected	187	158	84%	29	16%	
General practitioner	71	44	62%	28	38%	
Follow-up/specialist	44	39	89%	5	11%	
Density						0.045^$^
ACR 1	11	10	91%	1	9%	
ACR 2	149	128	86%	21	14%	
ACR 3	84	60	71%	24	29%	
ACR 4	27	21	78%	6	22%	
Missing	31	22	71%	9	29%	
BI-RADS score						< 0.001^$^
3	4	4	100%	0	0%	
4	4	2	50%	2	50%	
4a	72	63	88%	9	13%	
4b	149	126	85%	23	15%	
4c	46	34	74%	12	26%	
5	27	12	44%	15	56%	
Size category						< 0.001^$^
< 10 mm	92	83	90%	9	10%	
10–19 mm	90	77	86%	13	14%	
≥ 20 mm	120	81	67%	39	33%	
Multifocal within a quadrant						0.014^$^
No	284	231	81%	53	19%	
Yes	18	10	56%	8	44%	
Calcifications						0.005^#^
No	27	16	59%	11	41%	
Yes	275	225	82%	50	18%	
ER receptor						0.130^$^
Negative (< 80%)	100	74	75%	26	26%	
Positive (80–100%)	197	162	82%	35	18%	
Missing	5	5	100%	0	0%	
HER2 neu receptor						0.471^$^
Negative (0,1,2)	191	152	80%	39	20%	
Positive (3)	103	81	79%	22	21%	
Missing	8	8	100%	0	0%	
First resection						< 0.001^#^
Wide local excision	202	174	86%	28	14%	
Ablation	100	67	67%	33	33%	

**p*-Value of comparison between upstaged and non-upstaged patients determined with the chi-squared test^#^ or the Fisher’s exact test^$^

## Appendix D

Validation in the institution cohort: ROC and calibration plot of the prediction model for the risk of upstaging to invasive breast cancer of patients with biopsy-proven DCIS; analyses were done in the institution cohort without re-estimation of the model

DCIS-upstage model:

Jakub model:
